# Sex, Body Mass Index, and Dietary Fiber Intake Influence the Human Gut Microbiome

**DOI:** 10.1371/journal.pone.0124599

**Published:** 2015-04-15

**Authors:** Christine Dominianni, Rashmi Sinha, James J. Goedert, Zhiheng Pei, Liying Yang, Richard B. Hayes, Jiyoung Ahn

**Affiliations:** 1 Department of Population Health, New York University School of Medicine, New York, New York, United States of America; 2 New York University Perlmutter Cancer Center, New York, New York, United States of America; 3 Division of Cancer Epidemiology and Genetics, National Cancer Institute, National Institutes of Health, Bethesda, Maryland, United States of America; 4 Department of Pathology and Laboratory Medicine, New York Veterans Affairs Medical Center, New York, New York, United States of America; 5 Department of Pathology, New York University School of Medicine, New York, New York, United States of America; 6 Department of Medicine, New York University School of Medicine, New York, New York, United States of America; University of Illinois at Urbana-Champaign, UNITED STATES

## Abstract

Increasing evidence suggests that the composition of the human gut microbiome is important in the etiology of human diseases; however, the personal factors that influence the gut microbiome composition are poorly characterized. Animal models point to sex hormone-related differentials in microbiome composition. In this study, we investigated the relationship of sex, body mass index (BMI) and dietary fiber intake with the gut microbiome in 82 humans. We sequenced fecal 16S rRNA genes by 454 FLX technology, then clustered and classified the reads to microbial genomes using the QIIME pipeline. Relationships of sex, BMI, and fiber intake with overall gut microbiome composition and specific taxon abundances were assessed by permutational MANOVA and multivariate logistic regression, respectively. We found that sex was associated with the gut microbiome composition overall (p=0.001). The gut microbiome in women was characterized by a lower abundance of Bacteroidetes (p=0.03). BMI (>25 kg/m^2^ *vs*. <25 kg/m^2^) was associated with the gut microbiome composition overall (p=0.05), and this relationship was strong in women (p=0.03) but not in men (p=0.29). Fiber from beans and from fruits and vegetables were associated, respectively, with greater abundance of Actinobacteria (p=0.006 and false discovery rate adjusted q=0.05) and Clostridia (p=0.009 and false discovery rate adjusted q=0.09). Our findings suggest that sex, BMI, and dietary fiber contribute to shaping the gut microbiome in humans. Better understanding of these relationships may have significant implications for gastrointestinal health and disease prevention.

## Introduction

The human gastrointestinal tract carries a structured microbial composition consisting of more than ten-fold the number of human cells in the body [1]. The gut microbiota plays an indispensable role in digesting food components and in host immunological response [1, 2]. Comprehensive genomic-based microbiome assessments have identified significant inter-individual variation in the composition of the human gut microbiome [3]. Increasing evidence suggests that gut microbiome variation is important in the etiology of gastrointestinal diseases [4, 5] including cancer [6, 7], as we recently reported [8]. However, significant knowledge gaps remain in our understanding of personal factors that influence gut microbiome composition. Better understanding of these personal factors and their relationships with the gut microbiome may have important implications for disease prevention.

There is increasing evidence that sex steroid hormone levels are associated with the human gut microbiome [9]; however, the relationships of personal factors associated with these sex steroids in relation to the human gut microbiome has not been studied comprehensively. An animal study recently reported sex-specific differentials in gut microbiome composition [10]. Adiposity, a source of sex steroids [11], may play an additional role in forming a sex-specific microbiome composition, as suggested by animal models and human weight-loss trials [12–15]. Dietary fiber, a major energy source for gut bacterial fermentation [16], and an influence on systemic estrogen levels [17], may also shape the gut microbiome. The fiber hypothesis is supported by short-term dietary interventions [18–22], but there is only sparse data evaluating the impact of long-term dietary fiber on the gut microbiome [23, 24]. Given the findings from these early studies, we hypothesize that sex, adiposity, and dietary fiber operate through similar mechanisms, including hormone mediated pathways, to modulate the human gut microbiome.

To better understand the determinants of the human gut microbiome, we assessed gut microbiome composition in fecal samples from 82 subjects in the control group of a case-control study of colorectal cancer [8]. We investigated the relationship of sex, adiposity, assessed by body mass index (BMI) [25], and dietary fiber, individually and jointly, to gut microbiome composition and distribution of specific taxa and found that sex, BMI, and dietary fiber were associated with differences in the human gut microbiome.

## Methods and Materials

### Study population

Samples and data were collected from control group participants in a case-control study of gut microbiome and colorectal cancer, as recently described [8]. Study participants were originally recruited at three Washington, D.C., area hospitals (National Naval Medical Center, Walter Reed Army Medical Center, and George Washington University Hospital) between April 1985 and June 1987, prior to elective surgery for non-oncological, non-gastrointestinal conditions, including orthopedic or neurosurgical procedures, hernia repairs, vascular procedures, and other general surgeries [26, 27].

We included 82 subjects for study among 94 participants in the original investigation with at least 100 mg of lyophilized fecal material available (described below), excluding subjects with less than 1000 microbial sequence reads and with missing or extreme caloric intake (≤500 or ≥5500kcal/day). None of the eligible subjects reported taking antibiotics during the year prior to being recruited into the study. The study was approved by the institutional review boards from New York University School of Medicine and National Cancer Institute and all participants provided signed written informed consent. All signed written consent forms are documented and stored at the National Cancer Institute.

### Demographic, lifestyle, and dietary fiber assessment

Demographic information on age, sex, height, weight, and race (White, Black, Hispanic, Asian or Pacific Islander) were ascertained by structured demographic questionnaires completed prior to hospitalization and treatment. BMI was calculated by dividing weight in kilograms by squared height in meters. Study participants were then classified as normal weight (<25kg/m^2^) or overweight or obese (≥25kg/m^2^) based on the WHO definition [28]. Diet intake was assessed using a self-administered 100 item food frequency questionnaire. Long term intake frequency and portion size were queried. Nutrient intakes were derived from frequency and portion-size responses from the food frequency questionnaire, in which nutrient values per portion were multiplied by the daily frequency of intake and summed across all relevant food items, using the US Department of Agriculture pyramid food group serving database [29]. Diet data was standardized by total calorie intake [30]. Total fiber (5.7–11.3, 11.3–14.5, 14.5–17.6, 18.0–38.7 g/day), fruit and vegetable fiber (1.6–6.5, 6.6–8.8, 8.9–11.7, 11.8–21.9 g/day), bean fiber (0–0.9, 0.9–1.3, 1.4–2.3, 2.3–10.7 g/day), and grain fiber (1.0–2.5, 2.8–3.5, 3.6–5.1, 5.1–9.7 g/day) were grouped into quartiles. For taxa-specific analyses, total and specific sources of fiber were treated as continuous values.

### Fecal sample collection

Prior to hospitalization and treatment, subjects were asked to collect fecal samples at home, over a two day period, and store the material in a plastic container, in a Styrofoam chest containing dry ice. Fecal samples were shipped to a USDA laboratory, where the two-day samples were pooled, lyophilized and stored at a minimum of -40°C in sealed, air-tight containers.

### 16s rRNA gene sequencing microbiome assay

We extracted DNA from the stored fecal samples using the Mobio PowerSoil DNA Isolation Kit (Carlsbad, CA) with bead-beating. As we reported previously [8], 16S rRNA amplicons covering variable regions V3 to V4 were generated using primers (347F-5′GGAGGCAGCAGTRRGGAAT′-3′ and 803R 5′-CTACCRGGGTATCTAATCC-3′) incorporating Roche 454 FLX Titanium adapters (Branford, CT) and a sample barcode sequence [31]. Amplicons were sequenced with the 454 Roche FLX Titanium pyrosequencing system following the manufacturer’s specifications. Laboratory personnel were blinded to personal factor status.

### Sequence data processing and taxonomic assignment

Multiplexed, barcorded sequencing data was deconvoluted. Poor-quality sequences were filtered based on sequences less than 200 or greater than 1000 base pairs, missing or mean quality score <25, or mismatched barcode and primer sequences. Chimeric sequences were removed with ChimeraSlayer [32]. Filtered sequences were binned into operational taxonomic units with 97% identity and aligned to fully-sequenced microbial genomes (IMG/GG GreenGenes), using the QIIME pipeline [33]. Blinded quality control specimens in all sequencing batches (38 aliquots from 9 unmatched parent study control subjects) had good reproducibility, with intraclass correlation coefficients of 0.90 for principal coordinate analysis, 0.84 for Shannon diversity index, and 0.43 to 0.59 for relative abundances of major phyla [8].

### Statistical analysis

The relationship of overall gut microbiome composition with personal factors (sex, BMI, and dietary fiber intake) was assessed by principal coordinate analysis (PCoA), based on the unweighted (qualitative) phylogenetic UniFrac distance matrix [34]. PCoA plots were generated using the first two principal coordinates (PCs), according to categories of personal factors. Adonis [35], which uses permutational multivariate analysis of variance (PERMANOVA), was used to test statistical significances of association of overall composition with personal factors. To control for confounding, we used Generalized Linear Regression where each PC was treated as the outcome and the personal factors were mutually adjusted. Richness and evenness were assessed by Shannon’s diversity index and evenness index [36], respectively, and Monte Carlo permutations were used for assessment of statistical significance [33].

Relationships relating specific taxa abundance to sex and BMI were assessed by Mann-Whitney-Wilcoxon test. To visualize relationships of specific sources of fiber (bean, fruit and vegetable, and grain) with gut microbiome taxa (genus), we developed a heatmap based on unsupervised classification of Spearman correlation coefficients (R package, gplots; R version 2.15.3). To control for confounding in these analyses, we used logistic regression, considering high *vs*. low taxon abundance (median value cut-off) as the outcome and fiber types as the predictors.

False discovery rate (FDR) adjusted q-values were calculated for comparisons of taxa [37]. To minimize the number of null hypotheses that had to be corrected for, we limited these analyses to the three major phyla, Actinobacteria, Bacteroidetes, and Firmicutes (at least 50% of samples positive, with at least 1% median abundance). All analyses were carried out using SAS, version 9.3 (SAS Institute, Cary, NC) unless otherwise specified.

## Results

Among the 82 subjects included for study, 62.2% were men and 85.4% were white (Table 1). Their age ranged from 30–83 years (58.4±13.2 [mean ± standard deviation]). This study population included 46.3% former smokers and 9.8% current smokers. Mean intake of total dietary fiber was 14.1 grams per day. From the 82 immediately frozen fecal samples, gut microbiome was assessed by multiplexed 16S rRNA gene sequencing with amplification of the hyper-variable V3 to V4 region. In total, 331,288 high-quality 16S rRNA sequence reads (4,040±1,843 [mean ± standard deviation] sequences per sample) were binned to operational taxonomic units and subsequently assigned taxonomy.

**Table 1:** Population Characteristics.

Characteristic	Total (N = 82)	Men (N = 51)	Women (N = 31)	p-value^1^
Age (Years, Mean±SD)	58.4±13.18	58.0±13.58	59.2±12.67	0.71
BMI (Kg/M2^2^, Mean±SD)	25.0±4.06	25.6±3.49	23.8±4.69	**0.03**
Race (%)^2^				0.35
White	85.4	88.2	80.6	
Black	12.2	7.8	19.3	
Hispanic	1.2	2.0	~	
Asian or Pacific Islander	1.2	2.0	~	
Smoking Status (%)				**0.04**
Never	43.9	35.3	58.1	
Former	46.3	56.9	29.0	
Current	9.8	7.8	12.9	
Sequence Batch (%)				0.50
1	34.1	57.1	64.8	
2	65.9	42.9	35.2	
Sources of Fiber (Estimated Daily, Mean±SD)				
Total Dietary Fiber (grams)	14.1±5.11	14.3±5.24	13.8±4.95	0.07
Bean(grams)	1.8±1.62	2.1±1.79	1.3±1.15	**0.003**
Fruit and Vegetables(grams)	9.4±4.22	8.6±3.89	10.7±4.46	**0.01**
Grain(grams)	3.9±1.63	3.8±1.62	4.0±1.69	0.82

^1^All characteristics were compared by sex using either Chi square or Mann-Whitney-Wilcoxon tests. All analyses were carried out using SAS software (version 9.3).

^2^Race was grouped as White and Other for Chi square test.

**Fig 1:**
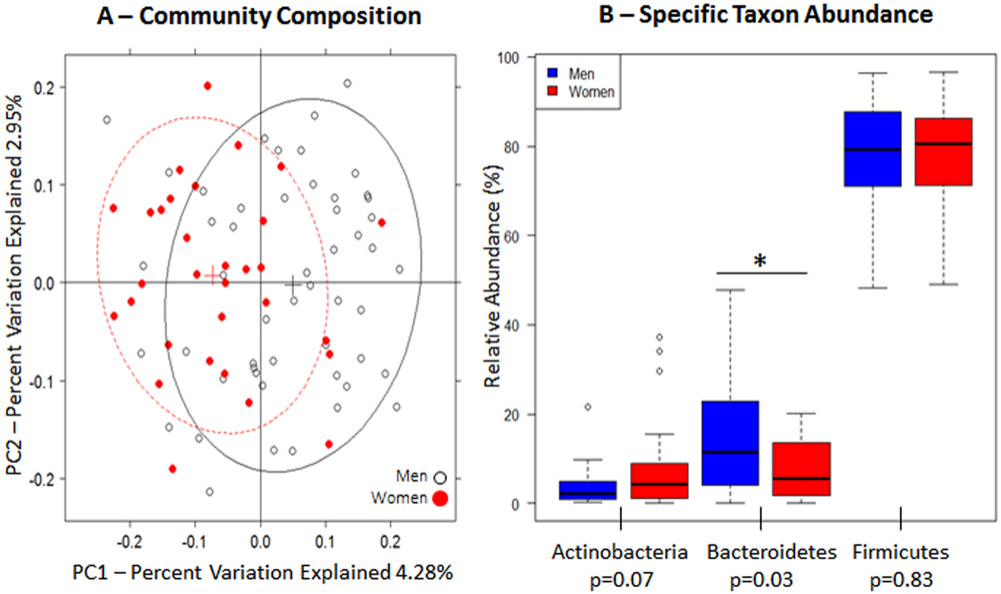
Gut microbiome according to sex. (A) Unweighted principal coordinate analysis plot of the first two principal coordinates categorized by sex. Ellipses were added to plots using the R package, latticeExtra (R version 2.15.3). (B) Relative abundance of the three major phyla. Mann-Whitney-Wilcoxon test was used to test for overall differences using SAS software (version 9.3). Nominal p-values are listed below each phylum.

Sex was significantly associated with gut microbiome composition overall (unweighted UniFrac p = 0.001, Fig 1A, Table 2); sex was specifically associated with the first principal coordinate, after we adjusted for other confounding factors (Table 3, p = 0.0001). Women tended to have a lower abundance of Bacteroidetes (5.5% in women [n = 31] *vs*. 11.4% in men [n = 51], FDR corrected q = 0.07) (Fig 1B).

**Table 2:** PERMANOVA^1^ analysis of personal factors with the unweighted UniFrac distance matrix.

Factor	F statistic	P-value
**Main Analysis**		
Sex	1.49	0.001
BMI^2^	1.12	0.05
Total Fiber^3^	1.08	0.14
Fruit & Vegetable Fiber^3^	1.13	0.06
Bean Fiber^3^	1.13	0.06
Grain Fiber^3^	1.08	0.15
**Men**		
BMI^2^	1.04	0.29
Total Fiber^3^	1.01	0.41
Fruit & Vegetable Fiber^3^	1.08	0.10
Bean Fiber^3^	1.17	0.01
Grain Fiber^3^	0.99	0.48
**Women**		
BMI^2^	1.17	0.03
Total Fiber^3^	1.26	0.005
Fruit & Vegetable Fiber^3^	1.13	0.05
Bean Fiber^3^	0.97	0.64
Grain Fiber^3^	1.19	0.01

^1^Adonis, which uses permutational multivariate analysis of variance (PERMANOVA), was used to test statistical significances of association of overall composition with personal factors. All analyses were carried out using the QIIME pipeline.

^2^BMI was categorized as normal weight (<25 kg/m^2^) versus overweight or obese (≥25 kg/m^2^).

^3^Total and specific sources of dietary fiber were categorized as low (quartiles 1–3) versus high (quartile 4) intake.

**Table 3:** Univariate and multivariate linear regressions^1^ for personal factors and unweighted principal coordinates (PC).

	PC 1 Estimate±SD	P-Value	PC 2 Estimate±SD	P-Value
**Individual Factors**				
Sex	-0.11±0.02	**4.81 × 10** ^**–5**^	0.01±0.02	0.76
BMI	-0.03±0.03	0.27	0.004±0.02	0.86
Fruit & Vegetable	-0.01±0.01	0.21	-0.01±0.01	0.36
Bean	0.01±0.01	0.45	0.004±0.01	0.72
Grain	0.01±0.01	0.39	-0.004±0.01	0.69
**Combined Factors** ^**2**^				
Fruit & Vegetable	0.003±0.01	0.82	-0.02±0.01	0.11
Sex	-0.10±0.02	**0.0001**	0.02±0.02	0.44

^1^ All estimates were computed using generalized linear regression models where principal coordinates were treated as outcomes and all personal factors treated as predictor variables. All analyses were carried out using SAS software (version 9.3).

^2^ Fiber from fruits and vegetables, sex, race, and age were included jointly in multivariate regression models.

**Fig 2:**
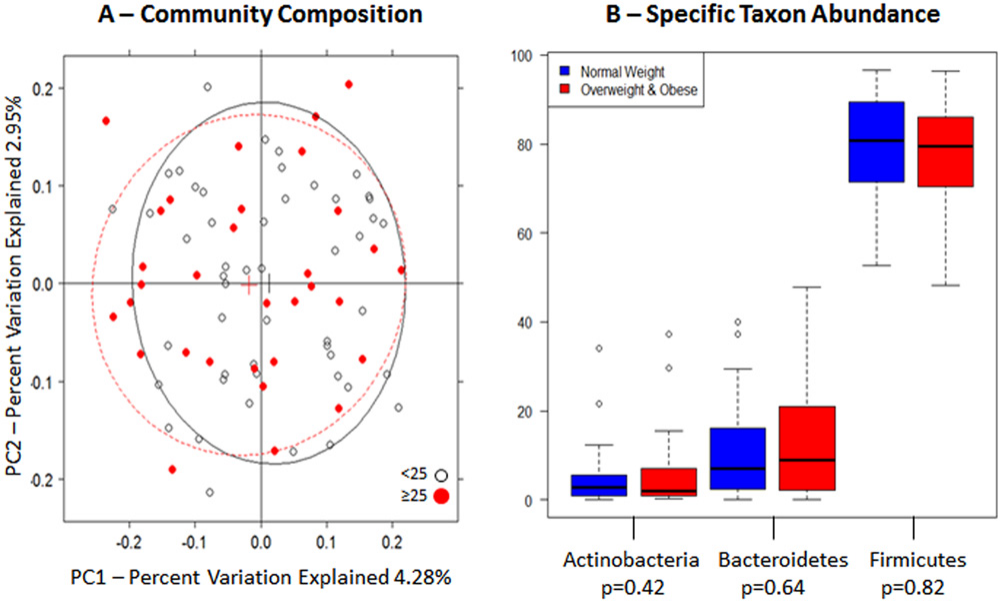
Gut microbiome according to BMI. (A) Unweighted principal coordinate analysis plot of the first two principal coordinates categorized by BMI (<25 kg/m^2^, ≥25 kg/m^2^). Ellipses were added to plots using the R package, latticeExtra (R version 2.15.3). (B) Relative abundance of the three major phyla. Mann-Whitney-Wilcoxon test was used to test for overall differences using SAS software (version 9.3). Nominal p-values are listed below each phylum.

**Fig 3:**
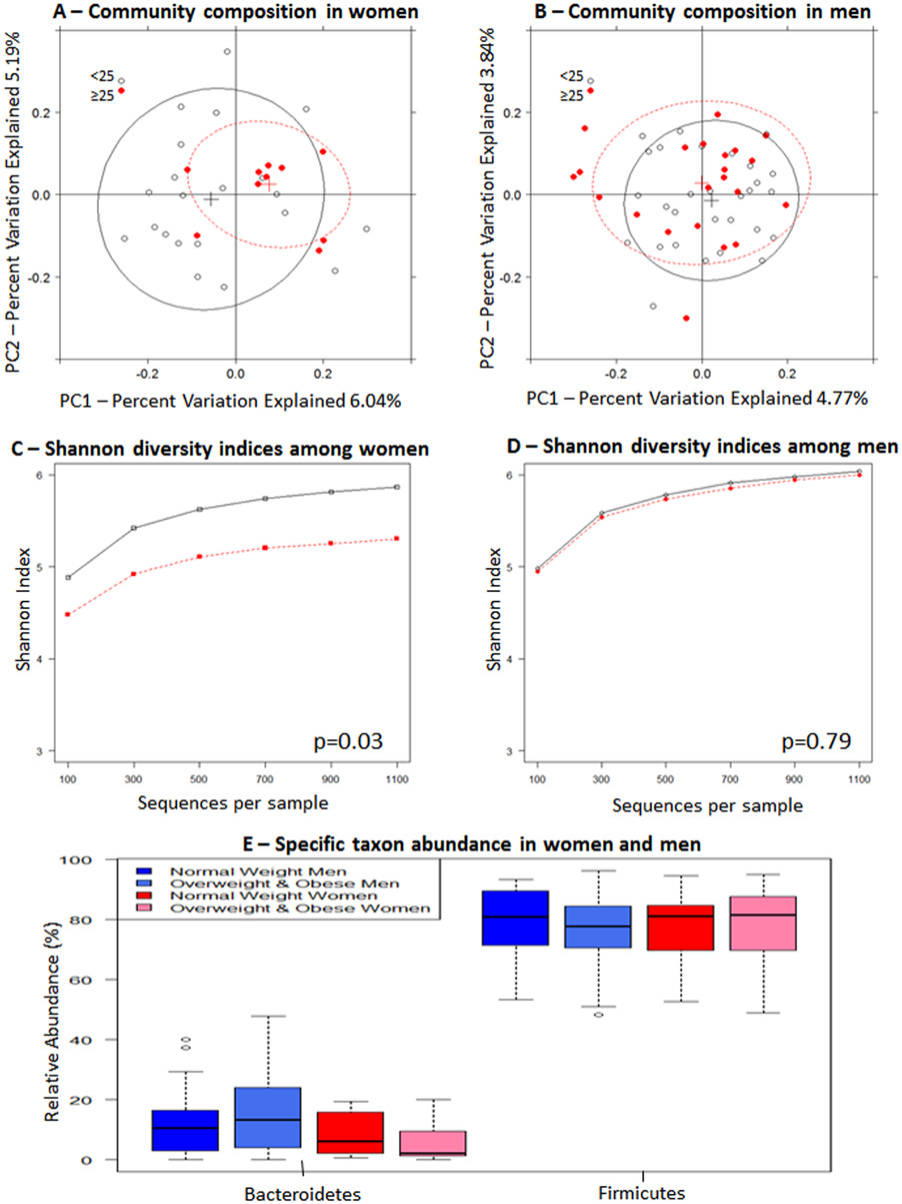
Gut microbiome according to BMI in women and men separately. Unweighted principal coordinate analysis plot of the first two principal coordinates categorized by BMI (<25 kg/m^2^, ≥25 kg/m^2^) in (A) women and (B) men. Ellipses were added to plots using the R package, latticeExtra (R version 2.15.3). Alpha rarefaction plots of Shannon diversity indices grouped by normal weight (<25 kg/m^2^; open circles) and overweight/obese (≥25 kg/m^2^; red circles) status for women (C) and for men (D). Statistical significance was assessed by non-parametric Monte Carlo permutations (QIIME). (E) Relative abundance of Firmicures and Bacteroidetes. Mann-Whitney-Wilcoxon test was used to test for overall differences using SAS software (version 9.3).

BMI (≥25 kg/m^2^ [n = 32] *vs*. <25 kg/m^2^ [n = 50]) was associated with gut microbiome community composition with marginal statistical significance (Fig 2A, p = 0.05, Table 2). When we stratified by sex, the relationship of BMI with overall gut microbiome composition was significant in women (p = 0.03, Fig 3A, Table 2) but not in men (Fig 3B, p = 0.29, Table 2). In women, Shannon diversity indices were lower in the overweight and obese subjects (n = 10) compared to normal weight subjects (n = 18) (Fig 3C, p = 0.03). In women, there was also a tendency for Bacteroidetes abundance to be lower in overweight and obese subjects, compared to normal weight subjects (Fig 3E).

**Fig 4:**
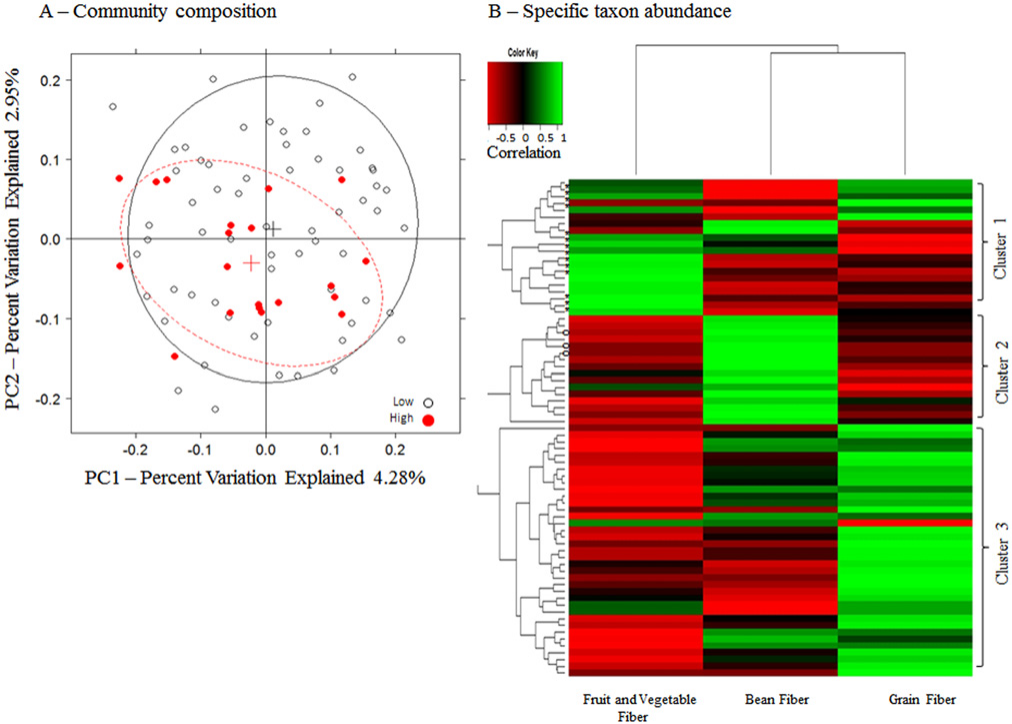
Gut microbiome according to dietary fiber intake. (A) Unweighted principal coordinate analysis plot of the first two principal coordinates categorized by fruit and vegetable fiber intake (Low: 1.6–11.7 g/day [equivalent to quartile 1–3], High: 11.7–21.9 g/day [quartile 4]). Ellipses were added to plots using the R package, latticeExtra (R version 2.15.3). (B) A heatmap based on unsupervised classification of Spearman correlations between the relative abundance of taxa (genus level) and three dietary fiber sources using the R package, gplots, (R version 2.15.3). For this analysis, only genera that were present in ≥15% of samples were included. Taxa belonging to Clostridia (Cluster 1, *) and Bifidobacteriales (Cluster 2, **°**) are marked.

Sources of dietary fiber intake tended to be associated with the gut microbiome. Fiber from fruits and vegetables (p = 0.06) and from beans (p = 0.06) were marginally associated with overall gut microbiome composition (Fig 4A, Table 2). The relationships of total fiber, fiber from fruits and vegetables, and fiber from grains were particularly stronger in women (p = 0.005, p = 0.05, p = 0.01, respectively, Table 2). Fiber from beans was strongly related to overall gut microbiome in men (p = 0.01, Table 2). Unsupervised classification showed that intake of fiber from fruits and vegetables clustered notably with class Clostridia (Cluster 1, Fig 4B, S1 Table). Multivariate logistic regression, adjusting for confounding, further supports this relationship (Table 4, OR for high *vs*. low Clostridia abundance = 1.24, p = 0.009 and FDR adjusted q = 0.09). We noted a second cluster of fiber from bean intake with members of Actinobacteria phylum (Table 4, OR = 2.24, p = 0.006 and FDR adjusted q = 0.05), and Bifidobacteriales order (OR = 1.65, p = 0.04 and FDR adjusted q = 0.43). No clear associations were observed between intake from grain fiber and microbial taxa (Table 2). Results of fiber intakes with specific microbial taxa were consistent in men and in women.

**Table 4:** Odds Ratio^1^ of having higher relative abundances of taxa by total and specific fiber intake.

	Total			Fruit & Vegetable			Bean			Grain		
	OR			OR			OR			OR		
	(95% CI)	p	q^2^	(95% CI)	p	q^2^	(95% CI)	p	q^2^	(95% CI)	p	q^2^
**Firmicutes**	1.19	0.46	0.46	1.09	0.20	0.60	1.14	0.42	0.66	0.90	0.45	0.66
	(0.75–1.90)			(0.96–1.24)			(0.83–1.57)			(0.68–1.19)		
Bacilli	1.01	0.87	0.87	1.00	0.97	0.97	0.97	0.85	0.97	1.05	0.72	0.97
	(0.90–1.10)			(0.90–1.13)			(0.70–1.31)			(0.80–1.39)		
Clostridia	1.15	0.03	0.12	1.24	**0.009**	**0.09**	1.09	0.58	0.97	1.06	0.68	0.97
	(1.01–1.20)			(1.05–1.47)			(0.08–1.49)			(0.79–1.42)		
Clostridium^3^	1.12	0.12	0.92	1.19	0.07	0.60	1.12	0.89	0.95	1.03	0.86	0.95
	(0.97–1.30)			(0.98–1.43)			(0.69–1.82)			(0.73–1.45)		
Blautia	1.04	0.49	0.92	1.02	0.83	0.95	1.56	0.02	0.51	0.88	0.40	0.85
	(0.94–1.15)			(0.88–1.17)			(1.07–2.27)			(0.65–1.19)		
Clostridium	0.95	0.28	0.92	0.89	0.09	0.64	1.43	0.06	0.60	0.80	0.13	0.74
	(0.86–1.04)			(0.78–1.02)			(0.98–2.07)			(0.59–1.07)		
Coprococcus	1.16	0.01	0.17	1.23	0.009	0.46	1.15	0.36	0.83	1.15	0.33	0.83
	(1.03–1.31)			(1.05–1.43)			(0.85–1.58)			(0.86–1.54)		
Dorea	1.01	0.86	0.92	1.00	0.99	0.99	1.64	0.03	0.51	0.95	0.72	0.95
	(0.92–1.11)			(0.87–1.14)			(1.05–2.57)			(0.71–1.26)		
**Bacteroidetes**	0.76	0.26	0.39	0.87	0.06	0.27	0.90	0.53	0.66	1.08	0.59	0.66
	(0.48–1.22)			(0.76–1.01)			(0.66–1.23)			(0.81–1.43)		
Bacteroidaceae	0.95	0.28	0.70	0.93	0.26	0.87	0.89	0.48	0.91	1.10	0.50	0.91
	(0.86–1.04)			(0.82–1.06)			(0.65–1.23)			(0.83–1.46)		
Porphyromonadaceae	0.89	0.04	0.40	0.89	0.08	0.60	0.58	0.02	0.57	0.96	0.77	0.91
	(0.80–0.99)			(0.78–1.02)			(0.37–0.91)			(0.73–1.27)		
**Actinobacteria**	1.36	0.22	0.39	0.97	0.74	0.74	2.24	**0.006**	**0.05**	1.17	0.29	0.65
	(0.83–2.25)			(0.86–1.11)			(1.27–3.96)			(0.87–1.59)		
Bifidobacteriales	1.01	0.82	0.84	0.97	0.62	0.92	1.65	0.04	0.43	1.12	0.46	0.86
	(0.92–1.12)			(0.84–1.11)			(1.02–2.68)			(0.83–1.52)		
Coriobacteriales	0.93	0.17	0.32	0.88	0.08	0.43	0.98	0.92	0.92	1.01	0.92	0.92
	(0.83–1.03)			(0.76–1.01)			(0.72–1.35)			(0.76–1.36)		

^1^All estimated OR’s and 95% CI’s were computed using multivariate logistic regression models where low and high relative abundances were treated as outcome and fiber intake treated as continuous variables of interest. Low and high groups were based on median value. Age, sex, race, and smoking status were controlled for. All analyses were carried out using SAS software (version 9.3).

^2^False discovery rate adjusted q-value.

^3^Taxa treated as absent/present because they were <80% present across all 82 samples.

## Discussion

In this study, we found that women had significantly different gut microbiome composition overall and particularly lower Bacteroidetes abundance, compared with men. BMI was associated with altered microbiome community composition. Greater intake of fiber from fruits and vegetables and from beans was associated with greater Clostridia and Actinobacteria abundances, respectively.

We identified significant sex differentials in gut microbiome composition and abundance of specific taxa. Our findings are consistent with an earlier targeted study of fluorescent *in situ* hybridization, reporting lower abundance of Bacteroidetes in women than men [38]. Furthermore, studies in mice showed that Bacteroidetes abundance is lower in female rodents [39], with sex differences in gut microbiome not appearing until after puberty [10, 39], supporting a role for hormonal and other host factors related to sexual maturity in shaping the gut microbiome. In addition to hormone-microbiome relationships, sex may impact the gut microbiome through alterations in gut transit time, which is reported to be greater for women than men [40, 41].

We found significant differences in gut microbiome composition by BMI particularly in women. Ley et al., reported that genetically modified obese mice carried less Bacteroidetes than lean mice [12] and later in humans, that weight-loss diets in obese subjects increased Bacteroidetes abundance [13]. Consistent with this, Turnbaugh et al. reported significantly reduced Bacteroidetes in obese compared to normal weight female twins [14]. Although mechanisms are unclear, adipose tissue is a source of estrogen production in obese individuals [11], and estrogen-microbiome associations have been suggested [9]; consistent with our findings of inter-relationships between sex, BMI, and gut microbiota composition. Our findings provide further support for a role of BMI in shaping the gut microbiome and points to potential sex-specific effects. Further study with larger subject numbers will help to elucidate the role of sex and BMI in influencing the gut microbiome composition. Furthermore, since obesity is a significant risk factor of many chronic diseases [52,53], including colorectal cancer [54], these findings may also point to potentially harmful microbes.

We found that specific sources of dietary fiber were differentially associated with the gut microbiome. Dietary fiber is a heterogeneous and complex mixture of different combinations of monosaccharides [42]. Fiber from fruit and vegetable intake was related to the gut microbiome composition, noted by an increased abundance of the Clostridia class; these findings are supported by two studies of long-term dietary fiber intake [23, 24]. Fiber may be enhancing gram-positive bacteria, including Clostridia [43], by reducing the gut transit time and pH [44, 45]. Clostridia are also an important class of dietary fiber fermenters where the major products are the potentially anti-carcinogenic short chain fatty acids [46, 47]. We recently reported significantly lower abundances of Clostridia in colorectal cancer patients compared to control subjects from this same study population [8]. Our observation here of an association between bean fiber intake and Actinobacteria abundance, particularly Bifidobacteriales, is supported by a short-term randomized trial which showed that fiber found in legumes, including beans [48, 49], increased the number of Bifidobacteria [19]. However, additional randomized, crossover studies have shown that concentrations of Bifidobacteria increase with other types of fiber treatments [18, 20, 21]. While the majority of studies are based on short-term randomized trials of high fiber supplements, our study adds to the growing body of evidence [23, 24] that usual long-term fiber intake influences the gut microbiome.

We also observed sex-specific effects on the relationship between dietary fiber intake and the gut microbiome. While mechanisms are not well understood, dietary fiber has been shown to influence the systemic levels of estrogen [17]. Dietary fiber is considered beneficial for human health [55] and is also an important energy source for gut bacterial fermentation [56]. Dietary fiber may be acting through sex hormone mediated pathways to influence the human gut microbiome.

Several limitations in our study should be considered. The study subjects are not representative of the general population, as they were awaiting elective surgeries for non-oncological and non-gastrointestinal conditions [8]. Measurement error is an inherent limitation in dietary intake assessment and may have attenuated the true relationship. We controlled in the statistical analysis for possible confounding factors, although other unmeasured factors may have influenced the observed relationships. For example, dietary fiber intake may mark for intake of foods that are rich in vitamins, minerals, and phytochemicals, or may be a marker of a healthy lifestyle. Stool samples, as used here, are not entirely representative of whole gut microbial composition [50], but obtaining alternative gut tissue biospecimens in healthy subjects is not feasible. Furthermore, archived stool samples may not be optimal compared to freshly collected samples, although our blinded quality control assay indicated good reliability (intraclass correlation coefficients = 0.84–0.90 for overall gut microbiome composition and 0.43–0.59 for major taxa) [8] and the identified major taxon distributions were within ranges of other published studies [51, 52]. We also cannot exclude the possibility that our findings may be due to chance because of the small sample sizes, particularly in our subset analysis.

This study has several strengths. The assessments on demographic, lifestyle, and dietary factors provide the ability to assess the relationship of these factors with the gut microbiome, while controlling for multiple comparisons. Complementing previous short-term intervention studies of high fiber supplement intake [18–22], this study provides useful information on associations of usual long-term daily fiber intake with differences in the gut microbiome in free-living people. Finally, our analysis of specific sources of fiber helps to better understand the fiber and gut microbiome relationship.

We found that sex, BMI, and source of dietary fiber are associated with differences in the gut microbiota. There is increasing evidence of a relationship between gut microbiota and gastrointestinal diseases [4, 5], including colorectal cancer [6–8], and other diseases, such as diabetes [53, 54]. These personal factors are important in an overlapping spectrum of chronic diseases [55–58] and may partly operate through common microbial pathways influencing hormone profiles [11, 17] and gut transit [44]; our findings may have implications for disease prevention.

## Supporting Information

S1 Table Genera and spearman correlation coefficients corresponding to Fig 4. (DOCX)
