# Supplementary material for: Sex, Body Mass Index, and Dietary Fiber Intake Influence the Human Gut Microbiome
Source: PLoS One. 2015 Apr 15;10(4):e0124599. doi: 10.1371/journal.pone.0124599 (PMC4398427; doi:10.1371/journal.pone.0124599)
Supplement: S1 Table — (DOCX) [file pone.0124599.s001.docx]

**Table S1.** Genera and spearman correlation coefficients corresponding to Figure 4

| Phylum | Higher-level Taxon (Genus) | Total Fiber^1^ | Fruit & Vegetable Fiber | Bean Fiber | Grain Fiber |
| --- | --- | --- | --- | --- | --- |
| Firmicutes | Catabacteriaceae (Unidentified) | 0.108 | 0.126 | 0.004 | 0.156 |
| Firmicutes | Ruminococcaceae (Eubacterium) | 0.156 | 0.123 | -0.022 | 0.145 |
| Firmicutes | Lachnospiraceae (Coprococcus) | 0.202 | 0.190 | 0.056 | 0.165 |
| Firmicutes | Ruminococcaceae (Anaerotruncus) | 0.165 | 0.057 | 0.066 | 0.176 |
| Actinobacteria | Coriobacteriales (Unidentified) | 0.107 | 0.109 | 0.061 | 0.105 |
| Actinobacteria | Unidentified | 0.240 | 0.169 | 0.157 | 0.197 |
| Actinobacteria | Coriobacteriaceae (Unidentified) | 0.084 | 0.072 | 0.150 | 0.026 |
| Firmicutes | Ruminococcaceae (Bacteroides) | 0.071 | 0.026 | 0.152 | 0.032 |
| Firmicutes | Clostridiaceae (Clostridium) | 0.142 | 0.124 | 0.100 | 0.002 |
| Firmicutes | Clostridia (Unidentified) | 0.235 | 0.321 | 0.114 | -0.140 |
| Firmicutes | Lachnospiraceae (Blautia) | 0.077 | 0.128 | 0.086 | -0.197 |
| Firmicutes | ClostridialesFamilyXIII.IncertaeSedis (Unidentified) | 0.212 | 0.227 | -0.013 | 0.049 |
| Firmicutes | ClostridialesFamilyXIII.IncertaeSedis (Eubacterium) | 0.144 | 0.197 | -0.050 | 0.036 |
| Firmicutes | Clostridiales (Unidentified) | 0.085 | 0.142 | 0.017 | -0.030 |
| Actinobacteria | Coriobacteriaceae (Adlercreutzia) | 0.079 | 0.094 | -0.040 | -0.055 |
| Actinobacteria | Coriobacteriaceae (Eggerthella) | 0.198 | 0.289 | -0.218 | -0.027 |
| Actinobacteria | Coriobacteriaceae (Unidentified) | 0.117 | 0.181 | -0.224 | -0.088 |
| Firmicutes | Ruminococcaceae (Clostridium) | -0.075 | -0.013 | -0.128 | -0.159 |
| Firmicutes | Lachnospiraceae (Unidentified) | 0.029 | 0.059 | -0.126 | -0.084 |
| Firmicutes | Clostridiales (Unidentified) | -0.038 | -0.006 | -0.097 | -0.052 |
| Firmicutes | Veillonellaceae (Dialister) | 0.001 | -0.019 | 0.193 | 0.079 |
| Actinobacteria | Bifidobacteriales (Unidentified) | -0.021 | -0.030 | 0.177 | 0.038 |
| Actinobacteria | Bifidobacteriaceae (Bifidobacterium) | 0.021 | -0.018 | 0.233 | 0.033 |
| Firmicutes | Ruminococcaceae (Faecalibacterium) | 0.000 | -0.126 | 0.232 | 0.007 |
| Actinobacteria | Bifidobacteriaceae (Unidentified) | 0.030 | -0.015 | 0.305 | -0.013 |
| Firmicutes | Veillonellaceae (Unidentified) | -0.077 | -0.048 | 0.142 | -0.048 |
| Firmicutes | Streptococcaceae (Unidentified) | -0.083 | -0.086 | 0.132 | -0.042 |
| Bacteroidetes | Rikenellaceae (Alistipes) | 0.006 | -0.023 | 0.079 | -0.038 |
| Firmicutes | Clostridiaceae (Unidentified) | -0.011 | -0.001 | 0.124 | -0.150 |
| Firmicutes | Veillonellaceae (Acidaminococcus) | -0.116 | -0.089 | 0.179 | -0.145 |
| Firmicutes | Streptococcaceae (Lactococcus) | -0.094 | -0.032 | 0.007 | -0.156 |
| Bacteroidetes | Prevotellaceae (Unidentified) | -0.133 | -0.087 | 0.004 | -0.108 |
| Firmicutes | Veillonellaceae (Unidentified) | -0.227 | -0.232 | 0.091 | -0.042 |
| Firmicutes | Lachnospiraceae (Dorea) | -0.094 | -0.107 | 0.077 | -0.059 |
| Firmicutes | ClostridialesFamilyXIII.IncertaeSedis (Unidentified) | -0.207 | -0.139 | 0.087 | -0.136 |
| Firmicutes | Lachnospiraceae (Clostridium) | -0.349 | -0.359 | 0.109 | -0.166 |
| Actinobacteria | Micrococcaceae (Rothia) | -0.268 | -0.312 | -0.295 | 0.113 |
| Firmicutes | Ruminococcaceae (Oscillospira) | 0.017 | 0.006 | 0.090 | 0.154 |
| Firmicutes | Lachnospiraceae (Unidentified) | 0.069 | 0.002 | 0.108 | 0.096 |
| Firmicutes | Lachnospiraceae (Lachnobacterium) | -0.058 | -0.053 | 0.139 | 0.127 |
| Firmicutes | Lachnospiraceae (Lachnospira) | 0.067 | -0.017 | 0.056 | 0.209 |
| Firmicutes | Lachnospiraceae (Eubacterium) | 0.011 | -0.028 | 0.032 | 0.136 |
| Firmicutes | Veillonellaceae (Veillonella) | -0.084 | -0.101 | 0.056 | 0.144 |
| Bacteroidetes | Bacteroidaceae (Bacteroides) | -0.060 | -0.120 | 0.026 | 0.117 |
| Bacteroidetes | Porphyromonadaceae (Odoribacter) | -0.080 | -0.117 | 0.009 | 0.109 |
| Firmicutes | Lactobacillaceae (Lactobacillus) | -0.079 | -0.097 | 0.076 | 0.064 |
| Actinobacteria | Coriobacteriaceae (Collinsella) | 0.048 | -0.019 | 0.044 | 0.076 |
| Actinobacteria | Bifidobacteriales (Unidentified) | -0.010 | -0.030 | 0.041 | 0.065 |
| Firmicutes | Ruminococcaceae (Ruminococcus) | 0.055 | 0.026 | 0.024 | 0.047 |
| Firmicutes | Lactobacillales (Unidentified) | -0.092 | -0.091 | 0.007 | -0.006 |
| Firmicutes | Unidentified | -0.062 | -0.018 | -0.018 | -0.032 |
| Firmicutes | Ruminococcaceae (Subdoligranulum) | -0.016 | -0.022 | -0.003 | 0.046 |
| Firmicutes | Turicibacteraceae (Unidentified) | -0.061 | -0.039 | -0.011 | 0.025 |
| Firmicutes | Turicibacteraceae (Turicibacter) | -0.018 | -0.052 | -0.058 | 0.018 |
| Firmicutes | Lachnospiraceae (Roseburia) | -0.036 | -0.120 | -0.060 | 0.110 |
| Bacteroidetes | Unidentified | -0.066 | -0.103 | -0.058 | 0.067 |
| Firmicutes | Bacilli (Unidentified) | -0.052 | -0.038 | -0.111 | 0.072 |
| Bacteroidetes | Rikenellaceae (Unidentified) | -0.050 | -0.071 | -0.127 | 0.082 |
| Bacteroidetes | Unidentified | -0.128 | -0.117 | -0.111 | 0.029 |
| Firmicutes | Carnobacteriaceae (Granulicatella) | -0.046 | -0.066 | -0.122 | 0.184 |
| Firmicutes | Veillonellaceae (Phascolarctobacterium) | -0.041 | -0.050 | -0.205 | 0.199 |
| Firmicutes | Ruminococcaceae (Unidentified) | 0.033 | 0.027 | -0.073 | 0.119 |
| Firmicutes | Ruminococcaceae (Unidentified) | 0.021 | 0.031 | -0.090 | 0.059 |
| Firmicutes | Peptostreptococcaceae (Tepidibacter) | 0.001 | 0.001 | -0.230 | 0.056 |
| Firmicutes | Streptococcaceae (Streptococcus) | -0.203 | -0.244 | -0.070 | 0.123 |
| Bacteroidetes | Unidentified | -0.146 | -0.217 | -0.108 | 0.115 |
| Bacteroidetes | Prevotellaceae (Prevotella) | -0.264 | -0.227 | -0.018 | -0.031 |
| Actinobacteria | Coriobacteriaceae (Slackia) | -0.168 | -0.154 | 0.000 | -0.046 |
| Bacteroidetes | Porphyromonadaceae (Unidentified) | -0.175 | -0.151 | -0.034 | -0.038 |
| Firmicutes | Gemellaceae (Gemella) | -0.139 | -0.102 | -0.082 | -0.057 |
| Actinobacteria | Actinomycetaceae (Actinomyces) | -0.180 | -0.169 | -0.097 | -0.050 |
| Firmicutes | Lachnospiraceae (Ruminococcus) | -0.151 | -0.208 | -0.139 | -0.020 |
| Bacteroidetes | Porphyromonadaceae (Parabacteroides) | -0.232 | -0.164 | -0.162 | -0.048 |

^1^Total fiber was included for comparison purposes although it does not appear in Figure 4.
